# Quantitative Analysis of the Corneal Collagen Distribution after* In Vivo* Cross-Linking with Second Harmonic Microscopy

**DOI:** 10.1155/2019/3860498

**Published:** 2019-01-10

**Authors:** Juan M. Bueno, Francisco J. Ávila, M. Carmen Martínez-García

**Affiliations:** ^1^Laboratorio de Óptica, Instituto Universitario de Investigación en Óptica y Nanofísica, Universidad de Murcia, Campus de Espinardo (Ed. 34), 30100 Murcia, Spain; ^2^Dpto. Biología Celular, Histología y Farmacología, Facultad de Medicina, Universidad de Valladolid, 47005 Valladolid, Spain

## Abstract

Corneal cross-linking (CXL) is a surgical procedure able to modify corneal biomechanics and stabilize keratoconus progression. Although it is known that CXL produces changes in corneal collagen distribution, these are still a topic of discussion. Here we quantitatively compare the corneal stroma architecture between two animal models four weeks after* in vivo* conventional CXL treatment, with second harmonic generation (SHG) imaging microscopy and the structure tensor (ST). The healing stage and the stroma recovery were also analyzed by means of histological sections. Results show that the CXL effects depend on the initial arrangement of the corneal collagen. While the treatment increases the order in corneas with a low level of initial organization, corneas presenting a fairly regular pattern are hardly affected. Histological samples showed active keratocytes in anterior and middle stroma, what means that the recovery is still in progress. The combination of SHG imaging and the ST is able to objectively discriminate the changes suffered by the collagen arrangement after the CXL treatment, whose effectiveness depends on the initial organization of the collagen fibers within the corneal stroma.

## 1. Introduction

Collagen cross-linking (CXL) is a minimally invasive technique based on a photochemical reaction carried out by a photosensitizer (riboflavin, vitamin B2) exposed to ultraviolet A light (UVA, 370 nm). During this operation, reactive oxygen species are produced, which induces covalent bonds [1] between collagen fibrils and fibers, and also between collagen and proteoglycans [2]. CXL has been reported to increase stromal stiffness [3, 4] and to be one of the most used clinical procedures to treat ectasias and halt keratoconus progression [3, 5, 6]. CXL has also improved other nonectatic disorders like infectious keratitis [7] and corneal edema [8].

CXL long-term effects have been analyzed in a number of clinical trials (both nonrandomized and randomized) [9–12]. Although most studies reported visual acuity improvements and stabilization of ectatic disease (with no alteration in transparency or reduction in keratometry values), the long-term efficacy of CXL procedures and their safety are still controversial. While some authors reported no progression during the follow-up [9, 10], others reported different failure rates [13–15].

Second harmonic generation (SHG) microscopy is a powerful nonlinear imaging technique used to visualize nonstained collagen-based structures, such as the corneal stroma [16]. SHG images of this stroma have provided information on fiber organization in healthy [15–17] and pathological samples, in particular keratoconic corneas [18–22]. Since CXL effects modify the collagen structure, SHG microscopy has been used to explore changes induced by CXL treatment. In particular, the organization of the collagen fibers was analyzed in CXL-treated rabbit eyes [23]. Two weeks after the treatment, the backward SHG signal showed markedly less waiving in treated areas compared to untreated ones. This fact was also observed by Bueno and coworkers in bovine and porcine eyes right after the treatment [24]. Another experiment analyzed forward and backward SHG images of rabbit corneas at different time intervals after CXL. No global differences in the fiber orientation and lamellar structure at any point time were reported [25]. In porcine corneas, forward SHG images showed that linear collagen fibers of control samples became wavy after CXL treatment [26]. In backward SHG images, treated porcine corneas presented an attenuated rugged/wavy pattern compared to the control group [27]. In a similar experiment (although comparisons with an accelerated CXL procedure were also made), CXL specimens were reported to present an increased fibrillar contrast in comparison with untreated baseline SHG images [28]. In a more recent study, SHG images of porcine corneas showed straighter fibers after CXL [29].

Although most of these experiments used a standard CXL protocol, the changes visualized in the SHG images are not completely coincident. Among others, the reasons might be the initial collagen organization of the different animal models [16], or the different experimental conditions of the involved samples (time after the CXL procedure, corneal buttons vs. entire ocular globes, etc.).

The changes detected in CXL corneas have mainly been analyzed in a qualitative way. Only a few explored the changes in the cornea morphology after CXL treatment in a quantitative manner [26, 27]. Tan and coworkers computed the degree of waviness [26]. This was not computed from the aspect ratio of the Fast Fourier Transform (FFT) elliptical fitting. Instead, they used the standard deviation of the angle distribution given by the FFT. Gupta and colleagues quantified the collagen architectural changes by means of an open-source software often used for image texture analysis [27]. They analyzed the roughness profile and computed the root mean square deviation (Rq) according to an international standardization texture parameter.

Although the FFT has been used to quantify stromal organization in healthy and pathological corneas [20, 22, 26, 29, 30], it might have some limitations for certain collagen distributions involving crosshatching, interweaving, and wavy patterns [31]. Alternative methods include the Radon transform [32], texture analysis [27], mathematical tensorial calculus [33], gray-level cooccurrence matrices [34], and the structure tensor (ST) among others [35].

A number of animal models with different stromal organizations have been employed to study the CXL effects. Results have been shown to depend on the sample and not to be conclusive. Moreover, to our knowledge, there is a lack of studies comparing morphological changes induced by CXL treatment in different animal models under similar experimental conditions. Two animal models (rabbit and chicken) presenting very different stromal organization [16] were involved in the present work. SHG microscopy was used to further analyze the changes suffered by the cornea after a standard CXL procedure. The stroma arrangement was analyzed at different depths after 4 weeks of treatment. The reason for the choice of this time point is based on previous results of corneal wound healing after surgery in both rabbits [6, 36, 37] and chickens [38, 39]. The ST was used as an objective tool to quantify the changes. In addition, histological analyses were also performed to explore and understand the clinical follow-up of wound healing after CXL treatment in those two animal models. Treated corneas were compared with control ones, and differences discussed.

## 2. Materials and Methods

### 2.1. Multiphoton Microscope

The SHG microscope setup used in this work was based on a custom instrument previously reported [24]. This combined a Ti:sapphire laser (Mira 900f, Coherent, St. Clara, CA) and an inverted microscope (Nikon TE2000-U). The illumination laser system operating at 76-MHz repetition rate was set to a wavelength of 800 nm. It passed through the XY scanning module and a dichroic mirror (used to separate the excitation light from the generated nonlinear signal) before being focused by a dry long working-distance microscope objective (Nikon ELWD, 20X/0.5 NA). The signal from the corneal tissues was collected in the backward direction through the same objective and filtered by a bandpass filter (FB400-10; Thorlabs Inc., Newton, NJ). This spectral filter isolated the SHG signal emitted by the corneal stroma of the specimens under analysis and avoided the detection of any other two-photon fluorescent signal. The SHG signal reached a photomultiplier tube used as detection unit. A DC-motor coupled to the objective was used for optical sectioning. Automatic recording procedure and image processing were carried out by custom-developed LabView™ and Matlab™ software.

### 2.2. Samples

Iber Braun adult chickens (N=12) and New Zealand adult rabbits (N=13) were used in this experiment. Animals were provided by Ibertec (Ibérica de Tecnología Avícola, S.A.U., Boecillo, Spain) and Granja San Francisco (Navarra, Spain), respectively. The entire experiment followed the guidelines of the Association for Research in Vision and Ophthalmology Statement for the Use of Animals in Ophthalmic and Vision Research. The use of tissue samples from animals and the protocol herein were approved by the Animal Ethics Committee of the Universities of Valladolid and Murcia.

### 2.3. Cross-Linking Treatment

The CXL treatment was performed in the left eye of both animal models (the fellow eye was used as control). The animals were anesthetized with intramuscular ketamine hydrochloride (Imalgene 500, Merial Laboratory S.A., Barcelona, Spain; 30 mg/kg), xylazine (Rompum, Bayer AG, Leverkusen, Germany; 5 mg/kg), and topical application of 0.5% tetracaine hydrochloride and 1 mg of oxybuprocaine (Colircusi Anestésico Doble, Alconcusí S.A., Barcelona, Spain). The corneal epithelium was firstly removed with a scalpel. Drops of riboflavin 0.1% solution in 20% dextran (Farmacia Magistral, Madrid, Spain) were instilled onto the cornea every 5 minutes for a duration of 30 minutes. Then, the cornea was irradiated with UVA light (370±5 nm and 3 mW/cm^2^ of irradiance) for 30 minutes by using a UV-X™ radiation system (IROC, Zurich, Switzerland). This corresponds to a total dose of 3.4 J or a total radiant exposure of 5.4 J/cm^2^. During the irradiation, the cornea was soaked with riboflavin/dextran solution every 5 minutes.

At day 30, animals were euthanized by intracardiac injection of sodium pentobarbital (Dolethal® 0737-ESP Vetoquinol, Madrid, Spain) under general anesthesia. Eyes were enucleated just after dead and the corneas excised by using ophthalmic scissors. A 2 mm scleral rim was left. Corneas were then fixed with 4% buffered paraformaldehyde for 24 h and rinsed in buffer phosphate (0.1 M). Manipulation and excision procedures were carried out by a well-experimented technician. All corneas appeared clear during the entire experiment. The CXL-treated corneas were compared to the control ones (fellow eyes).

### 2.4. SHG Imaging Procedure

These nonstained samples were placed on a glass bottom dish (thickness: 170 *μ*m) filled with buffer phosphate for SHG imaging. For each corneal location, three sequential SHG images (210x210 *μ*m^2^) corresponding to the central cornea (i.e., the corneal apex) were acquired. Every final SHG image was the average of those three individual frames. This simple image processing optimizes the signal-to-noise ratio. Along the Z-direction, images were acquired at different depth locations (20 *μ*m apart) across the entire corneal depth. In this work, the 0 *μ*m corneal depth position was established as the first location within the stroma where the SHG signal appeared (i.e., approximately Bowman's layer). Throughout this work, anterior (or outer), mid, and posterior (or inner) cornea refer to the areas of the stroma located within the corresponding thickness (i.e., 1st, 2nd, and 3rd, resp.). For instance, for a 240 *μ*m thick cornea, the anterior stroma corresponds to corneal layers located between Bowman's layer and an 80 *μ*m depth plane.

### 2.5. Analysis of the Collagen Distribution in SHG Images

**Figure 1:**
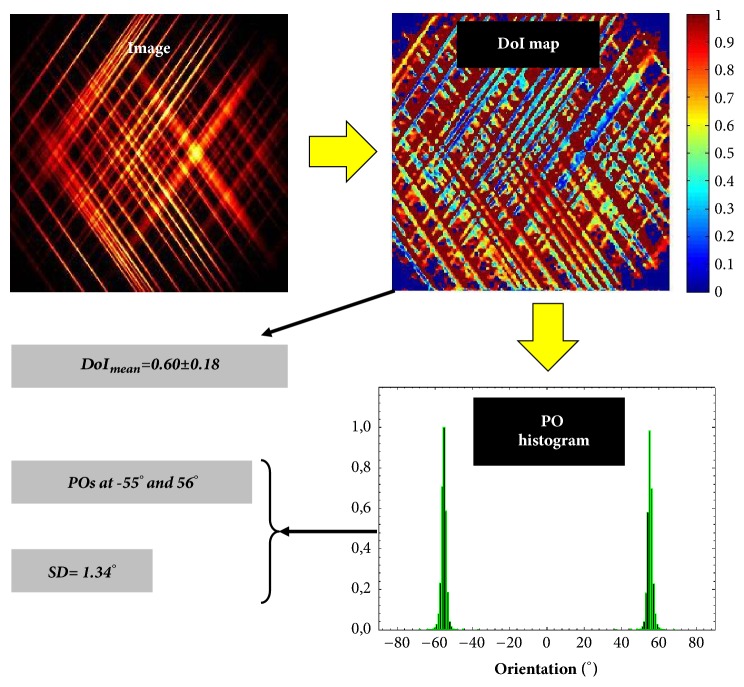
Example of the use of the ST in an artificial image presenting a structural organization with two preferential directions. The PO histogram represents the frequency of appearance of a certain orientation within the fibers (i.e., the closer to 1, the higher the presence of that direction in the image).

For each final SHG image, the total intensity was computed and the ST applied. The ST is a mathematical tool based on partial derivatives that provides information on the isotropy and preferential orientation of spatially resolved structures (pixel-by-pixel). Further technical details on this tool have been extensively reported in [35]. In particular, three parameters are computed: the degree of isotropy (DoI), the histogram of preferential orientation (PO), and the structural dispersion (SD) of the collagen fibers. As a general idea, DoI ranges between 0 and 1, and the higher DoI, the higher the alignment of the collagen fibers along a PO. If DoI is close to zero (or alternatively, a PO does not exist), collagen fibers present a nonorganized distribution. SD is defined as the standard deviation of PO histogram. Since DoI and SD have been shown to be linearly correlated (i.e., the higher the DoI, the higher the SD), we only take into account DoI values here. As an illustrative example, Figure 1 presents a schematic diagram showing the use of the ST in an artificial image.

To quantitatively differentiate the stromal arrangement between control and post-CXL corneas, the statistical analysis was carried out in three datasets: PO histograms, SHG total intensity, and DoI parameter. PO histograms were analyzed and compared using the Kolmogorov-Smirnov nonparametric test of the equality of continuous, avoiding making assumptions about the data distribution. This analysis was performed using the IBM SPSS Statistics software and differences were considered as significant when p-value was smaller than 0.05. The analysis concerning SHG intensity and DoI was carried out applying the t-test analysis (p<0.05) provided by OriginLab® software.

### 2.6. Bright-Field Microscopy

After the SHG imaging operation, the corneas were dehydrated through a series of graded ethanol and infiltrated with melted wax. Then, the tissues were sectioned using a microtome Minot (HM325 Microm, Spain) and sections (5 *μ*m thick) were refixed in Bouin's solution overnight. After removing the paraffin, these sections were stained with Masson's trichrome. This is a three-colour staining protocol used in histology that includes a sequence of three solutions: Weigert's iron hematoxylin for 10 min, Biebrich scarlet-acid fuchsin for 10-15 min, and light green for 5 min. Histological sections were examined under a bright-field microscope (Zeiss Axiophot HBO-50, Carl Zeiss, Jena, Germany). Photomicrographs were taken using the AxioCam Digital Camera and AxioVision Microscope software provided with the commercial microscope. The analysis of these histological sections will be useful to assess the depth of the affected corneal tissue and the posttreatment healing following the CXL procedure.

## 3. Results

### 3.1. SHG Imaging of Nonstained* Ex-Vivo* Corneas

**Figure 2:**
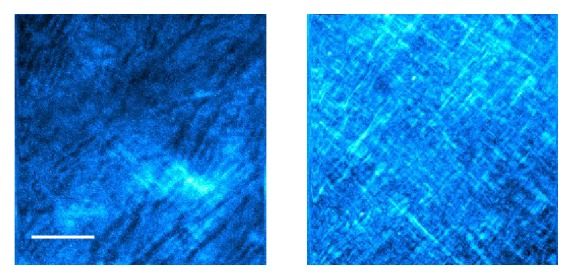
SHG signal from the corneal stroma in untreated rabbit (left) and adult chicken (right) corneas. Depth locations were 40 and 120 *μ*m, respectively. Bar length: 50 *μ*m.

Figure 2 shows SHG images corresponding to two control corneas (rabbit and adult chicken). Imaged areas correspond to randomly chosen planes. As expected, the collagen arrangements of both species are noticeably different [16]. Although collagen fibers are clearly outlined and run parallel to the corneal surface, differences in lamellar organization, thickness, and spacing are easily observed. In particular, for the rabbit cornea, adjacent fibers present overall similar orientations. However, the SHG image of the adult chicken shows longer fibers with orthogonal interweaving.

**Figure 3:**
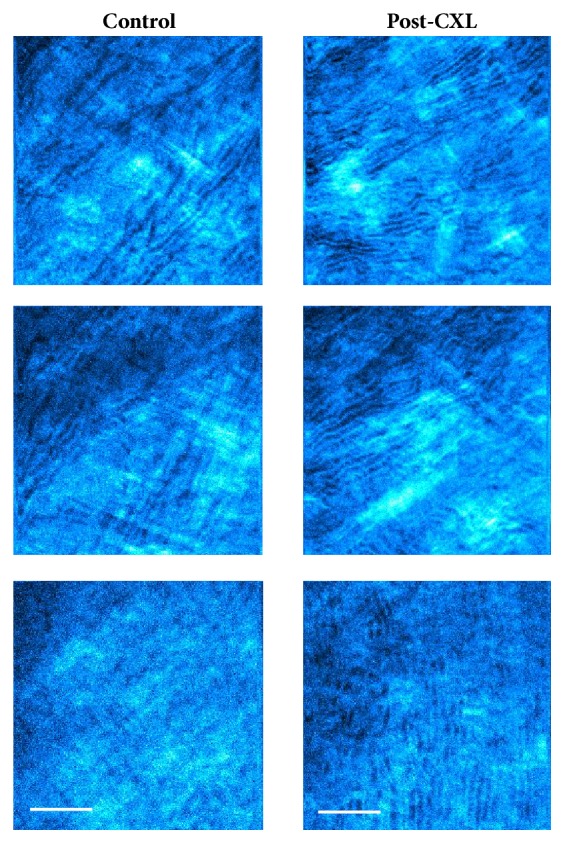
SHG images of a control rabbit cornea (left panels) and a post-CXL cornea (right panels). Depth positions correspond to the anterior (40 *μ*m, top), mid (120 *μ*m, middle), and posterior (240 *μ*m, bottom) locations. The size of the images is the same as in the previous figure. Scale bar: 50 *μ*m.

**Figure 4:**
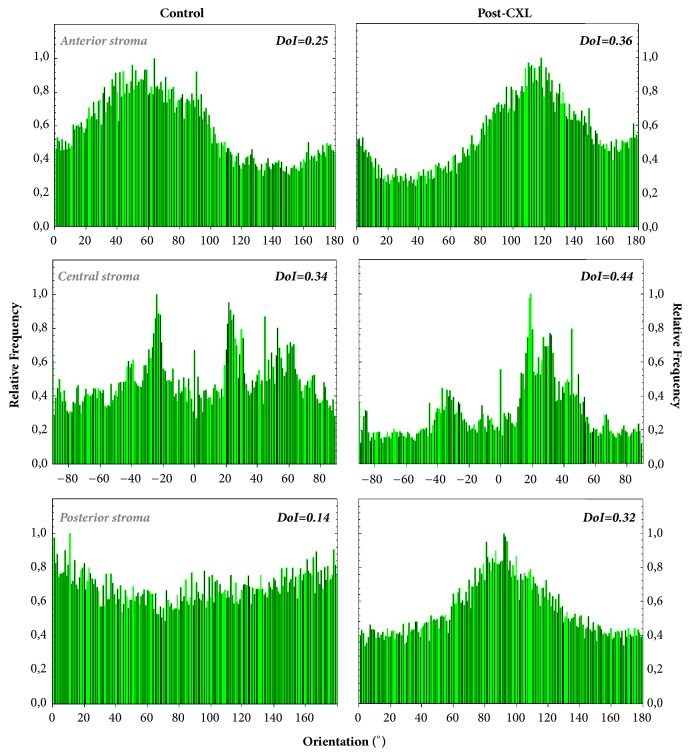
Histograms of PO distribution for a control and a post-CXL rabbit cornea at three depth locations. These were computed by applying the ST to the SHG images in Figure 3. The corresponding DoI mean values are also included for direct comparisons.

Figure 3 compares the SHG images of two rabbit corneas at different depth locations (anterior, middle, and posterior stroma), one control (left column), and one after four weeks of the* in vivo *CXL treatment (right column). From a qualitative point of view, it is difficult to comment on the changes caused by the CXL procedure in the arrangement of the collagen fibers. In general, and compared to the intact tissue, the collagen bundles appeared more delineated and less interwoven four weeks after CXL treatment. However, for a better understanding and description, a quantitative analysis must be carried out. In that sense, Figure 4 depicts the histograms of PO as well as the DoI values for the SHG images of Figure 3.

For both control and post-CXL corneas, the collagen distribution in the anterior and middle stroma presents POs indicated by the maximum values in the histograms. For both locations, the averaged DoI values are associated with a partially aligned collagen distribution (i.e., 0.20<DoI<0.70) [35]. However, this behavior differs at the posterior stroma. While the control cornea presents a nonorganized arrangement (i.e., DOI<0.20 and absence of PO), the post-CXL one turns into a partially organized structure (with a DoI clearly higher than 0.20). This fact was found for all the rabbit corneas involved in the experiment. It is also worth noticing that, for all depth locations, DoI values are higher in post-CXL corneas than in control ones. This indicates an increase in the organization of the corneal lamellae as a result of the CXL. These differences are emphasized by the fact that PO distributions for both experimental conditions were significantly different as computed by the Kolmogorov-Smirnov test (p<0.05). This means that the CXL treatment modifies the collagen structure across the entire cornea.

**Figure 5:**
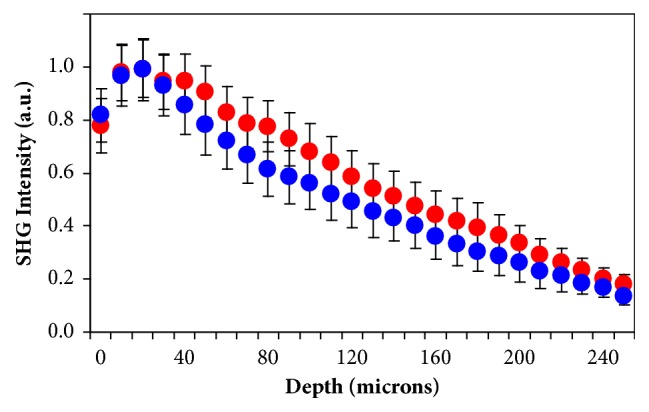
SHG intensity profiles as a function of depth for control (blue dots) and post-CXL (red dots) rabbit corneas. The data of each line are the average for all the specimens. The values have been normalized for a direct comparison.

The SHG intensity profiles as a function of depth for control and 4 weeks after CXL corneas are presented in Figure 5. The results correspond to the mean across all specimens for each experimental condition. It can be observed that the SHG intensity is higher in post-CXL corneas (17% on average). Moreover, these differences in the intensity profiles have been found to be statistically different (t-student, p=0.025).

**Figure 6:**
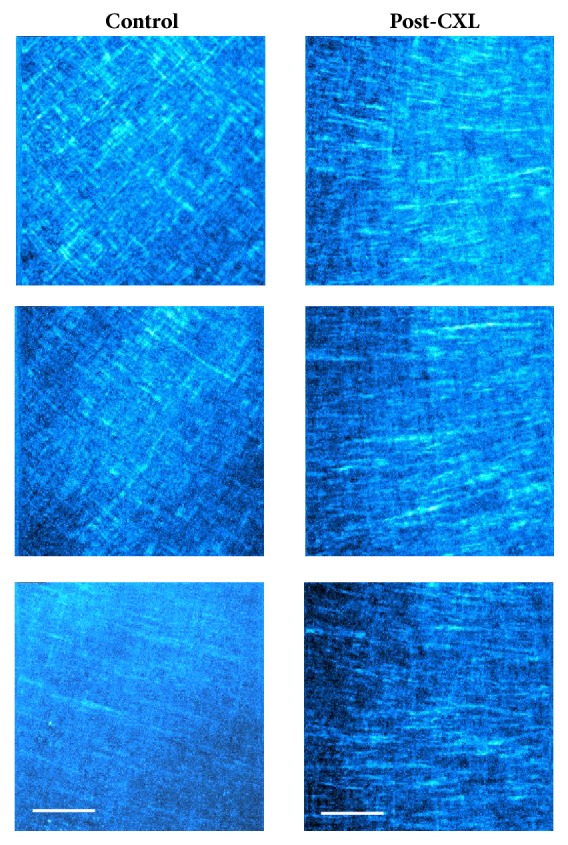
SHG images of a control adult chicken cornea (left panels) and a post-CXL cornea CXL (right panels). Depth positions (from top to bottom) correspond to the anterior, mid, and posterior locations. The size of the images is the same as in Figure 3.

For the chicken model, representative SHG images revealing the stroma in control and post-CXL corneas as a function of depth are depicted in Figure 6.

By simple visual examination, the presence of orthogonal interweaving is apparent at the anterior cornea and middle cornea. This seems to be less evident at the posterior location. For the treated eyes, the structure of the stroma shows long collagen fibers running parallel to each other.

**Figure 7:**
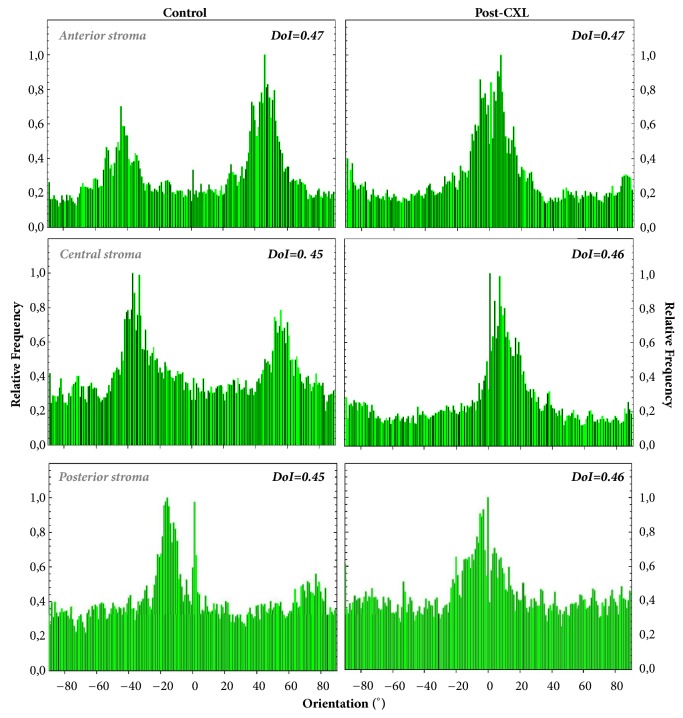
Histograms of PO distribution for an untreated (left) and a post-CXL chicken cornea (right) at three depth locations. The corresponding averaged DoI values are also included.

The corresponding PO distribution histograms of the previous SHG images are presented in Figure 7. For the control cornea, the anterior and midstroma present a fairly well-organized structure with two remarkable POs, located about 90° apart.

As expected from the corresponding SHG image, at the posterior stroma, one of the POs is much less pronounced and almost disappears. As a result of the CXL and posterior wound healing, for the treated cornea, only one PO is maintained. This finding was similar for all the chicken samples involved in the experiment. For both control and post-CXL corneas, DoI values always corresponded to partially aligned distributions. Changes in DoI after the treatment were not found at any corneal location. Moreover, it is interesting to notice that for this animal model the differences between pairs of PO histograms were not significant (Kolmogorov-Smirnov test, p>0.05 for all specimens and stroma locations). This indicates that the CXL treatment for the adult chicken cornea was not as efficient as it was in the rabbits.

**Figure 8:**
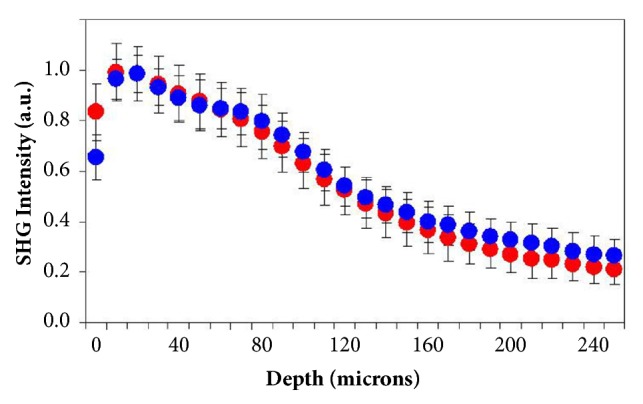
Comparison of SHG signal profiles as a function of corneal depth in control (blue dots) and post-CXL (red dots) chicken eyes. Each line corresponds to the mean values across all the specimens for each experimental condition. Data have been normalized for direct comparisons.

In Figure 8, a direct comparison of the SHG intensity profiles as a function of corneal depth is shown for the chicken model. Unlike in rabbit eyes, the signals for chickens was similar in control and post-CXL corneas. In addition, differences were not statistically significant (t-student, p=0.387).

For a better understanding of the CXL effects and the changes (if so) produced in the collagen distribution of both animal models, the DoI values provided by the ST (which inform on the collagen arrangement) have been explored more in depth.

**Figure 9:**
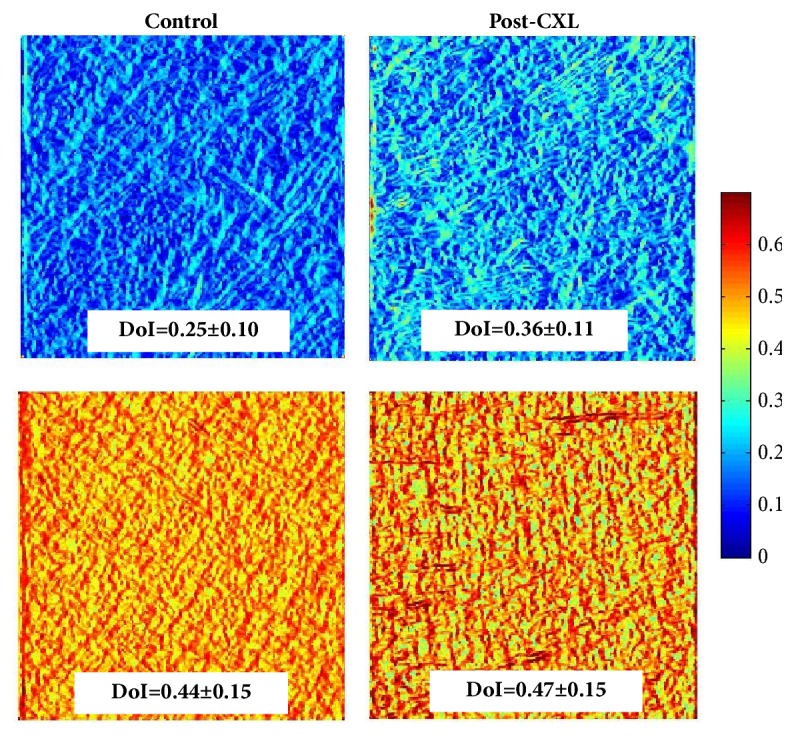
Representative DoI maps for post-CXL and control corneas (anterior stroma) for a rabbit (top) and an adult chicken (bottom) specimens. The averaged DoI value across each map is also given.

**Figure 10:**
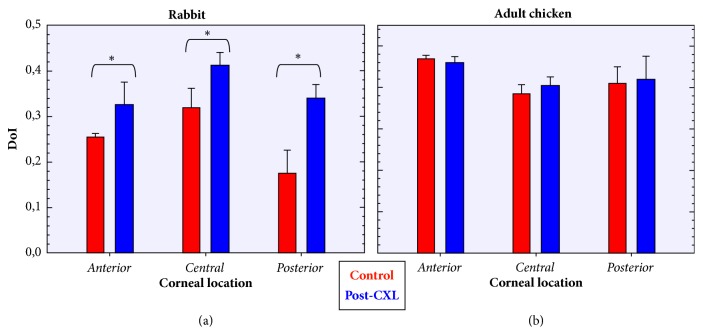
Comparison of averaged DoI values for control (red bars) and post-CXL (blue bars) corneas for three corneal locations. Data correspond to the mean values across all specimens (rabbits (a) and chickens (b)). Error bars indicate the standard deviation.

Figure 9 depicts four representative DoI maps of control and CXL-treated corneas in a rabbit (upper panels) and an adult chicken (bottom panels). The locations correspond to the anterior stroma. The inserted DoI values are the mean across the entire DoI map. In the rabbit, it can be observed that the DoI map for the CXL cornea is lighter than that of the control one. The differences in DoI values between both experimental conditions were positive for any location across the stromal thickness, which indicates that the CXL treatment increased the order of the lamellar structure (see Figure 10 for direct numerical comparisons). Unlike the rabbit model, the chicken cornea DoI maps provided similar values in control and post-CXL corneas (independently of the stroma location, Figure 10). This means that for this animal model the CXL treatment hardly affected the isotropy of the collagen fiber distribution.

For all the specimens involved in the experiment, Figure 10 depicts the averaged DoI values for the three locations in control and CXL-treated eyes. For each corneal location, the bars represent the mean across all animals (and corneal layers within that location). For the three locations, the DoI values of the rabbit corneas (Figure 10(a)) differed significantly (p<0.05) when comparing control and treated eyes. This means that the CXL treatment modifies the stroma arrangement leading to a higher organization. The data for the chicken corneas are presented in Figure 10(b). For those corneas, differences between both experimental conditions were not significant, which implies that the CXL treatment has little effect on the corneal collagen order in chickens.

### 3.2. Bright-Field Imaging of Stained Corneal Histological Sections

**Figure 11:**
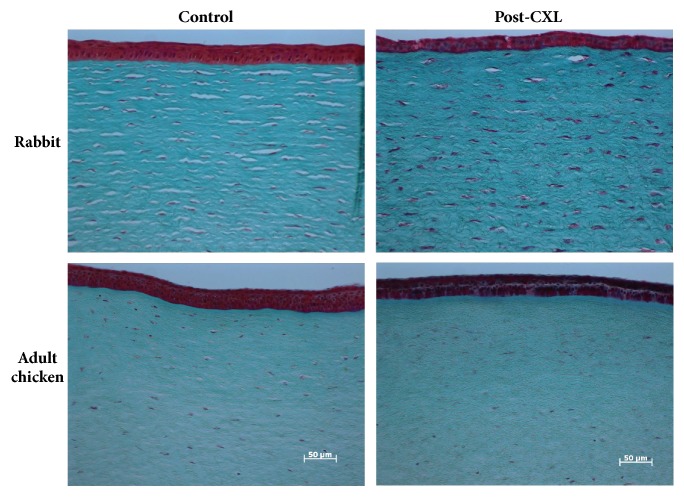
Histological sections of rabbit and chicken corneas (control vs. post-CXL) stained with Masson's trichrome. Dark stained cells are keratocytes. Activated keratocytes present a bigger size (see text for further information).

The specimens used for SHG imaging were also analyzed with a bright-field microscope once the corresponding histological sections were prepared (see Methods above).  Figure 11 shows representative photomicrographs of control and post-CXL corneas for the two animal models. It can be observed that, four weeks after the treatment, the epithelium has recovered. This is coherent with recent results by Lorenzo-Martín and coworkers [40]. Both control and post-CXL samples showed similar epithelium thickness as well as a homogeneous colour of the extracellular matrix. In post-CXL rabbit and chickens, an important depletion of keratocytes was produced in the anterior and middle stroma that is nearly recovered in the middle stroma after 30 days. In the anterior stroma, a depleted band still appeared just below the epithelium. Numerous activated keratocytes [41, 42] can also be seen across the entire stroma. These activated cells indicated that the anterior and middle stroma was compromised after CXL treatment. Moreover, the depletion region is associated with a “still in progress” wound healing (i.e., noncomplete healing at that temporal point) as also observed in a previous publication [40]. Stromal cells from chicken corneas displayed similar compromise; however, the wider depleted strip of keratocytes below the epithelium indicated a slower healing process (compared to the rabbit). In both animals, the endothelium was not damaged.

## 4. Discussion and Conclusions

In this work, we have investigated the performance of a standard CXL treatment in two animal models (rabbits and adult chickens) and the ability of SHG imaging microscopy to detect changes produced in the corneal stroma four weeks after the* in vivo* treatment. The results were compared with those of nontreated control corneas (contralateral eyes).

Although CXL treatment has been reported to be a very efficient clinical procedure in keratoconus human eyes, the effects at microscopic scale and the changes in collagen arrangement are still under discussion. Some authors compared the effects of using riboflavin alone or irradiating UVA without riboflavin instillation on the corneal stroma by analyzing TPEF signals [23, 43–45]. Unlike those experiments, our interest here was to explore the effects of the complete standard CXL procedure four weeks after the treatment.

In order to understand the effects of CXL, SHG imaging has been used in different animal models in the past. However, due to the broad experimental conditions of the corneas involved (initial stromal collagen distribution, reduced number of specimens, time after CXL, buttons vs. entire globes, fresh vs.* ex vivo*, etc.), direct comparisons cannot be done. This has been overpassed in the present work by using the same experimental conditions in two animal models. Here, the spatial distribution of the stroma fibers after four weeks of CXL has been compared with control specimens at different corneal depth locations.

Although CXL treatment modifies the arrangement of the collagen fibers, these are not always readily observable by a simple visualization. In that sense, a quantitative characterization was carried out by using the ST. Our results show that the response of corneas to CXL treatment differs between both animal models. In rabbit corneas, CXL provided a statistically significant increase in collagen organization as assessed by the DoI parameter (see Figures 4 and 10). This occurred for the different depth locations but it was more noticeable at the posterior cornea, where the collagen architecture was not organized (DoI<0.20). This global increase in collagen organization after CXL was corroborated when comparing the SHG profiles between control and treated corneas (Figure 5). As previously reported [46, 47], the more organized the collagen-based tissues, the higher the SHG signal (17% for the samples here involved). Moreover, SHG signal profiles were statistically different.

The microscopic corneal structure in birds is very different from that of mammals [48]. In particular, it has been shown here that collagen in control chicken corneas is better organized than in rabbits (see DoI values in Figure 7). The collagen arrangement in the control corneas of these two animal models agrees with previously reported data [16, 25, 49, 50]. In rabbits, the partial organization decreases at deeper locations. In the chicken cornea, the collagen bundles present an orthogonal interweaving organized distribution (Figure 7). This pattern shows a rotational shift with depth at the anterior and middle stroma that disappears at posterior locations where the PO is maintained [16, 49]. After CXL treatment, the anterior and middle stroma tend to present a unique PO (instead of two). However, the overall degree of organization is mostly maintained, probably due to the fairly good fiber organization of the initial conditions. At the posterior stroma, a single PO is present and CXL hardly affects the DoI. To the best of our knowledge, chicken corneas have not been previously used in SHG analysis of CXL treatments. Due to this, comparisons with previous reports cannot be done.

Corneal biomechanics is heavily influenced by the stromal collagen architecture [49]. Then, particular collagen distributions (as those here presented) might reflect a different biomechanical behaviour. It has also been reported that the degree of collagen fiber intertwining is linked to local stromal elastic modulus [48]. Then, a more organized stroma could be associated with a higher stiffness and vice versa. This indicates that, in regular conditions, chicken corneas might be more rigid than rabbit ones. This is coherent with keratoconic corneas presenting both a nonorganized structure and “higher flexibility.” Then, CXL treatment is very effective in those corneas probably due to their nonorganized stromal architecture. This fact would be in agreement with the present results: chicken corneas are hardly affected by CXL due to their well-organized structure.

The present work was exclusively centered on SHG imaging; however, further experiment involving biomechanical and CXL-induced stiffening measurements would be of interest to complete these results [40, 51–54].

Steven and colleagues reported a similar experiment to the one herein, but no quantitative data were provided. They analyzed rabbit corneas after two weeks of CXL treatment using SHG and two-photon fluorescence images [23]. The autofluorescent signal was markedly stronger in treated eyes (a similar finding was reported in [25]). Moreover, control eyes showed a regular wave-like orientation of collagen fibers that turned into a homogeneous pattern after CXL.

Our results are not completely coincident with those ones, probably due to the differences in the experimental conditions. While we used excised corneas, those authors employed entire ocular globes. Since intraocular pressure is released during this operation, some noncontrolled artefacts might be introduced [28, 50]. In addition, they claimed that the CXL effects were similar across the cornea, but only images up to the midstroma were used. We found some changes at those locations, but they were more evident at deeper locations.

The experiment carried out here is very different from the previously reported by one of the authors of this work [24]. In that previous experiment, both TPEF and SHG images were acquired to qualitatively analyze* ex vivo* post-CXL corneas (porcine and bovine) right after the CXL procedure and during a maximum of 2 hours. Short-time changes in corneas treated with riboflavin (but not followed by UV irradiation) were also explored. Here, the CXL treatment was carried out in* in vivo* conditions and the animals were sacrificed one month after the procedure. This permits the analysis of both CXL effects and wound healing response by means of SHG imaging.

Krüger and colleagues explored bidirectional SHG images of trephined rabbit corneal buttons 3 days, 6 days, and 6 weeks after CXL treatment (although an analysis with time was not given) [25]. Backward SHG images showed a slight loss of structure at the anterior cornea and the rest of corneal locations were similar to those of the control eyes. They conclude that, in general, CXL does not reflect major structural changes but they might be located at molecular levels well below the resolution limit of SHG microscopy (both forward and backward). Those findings do not agree with ours probably due to the absence of a quantitative analysis of their SHG images. A simple visual analysis of SHG images might also lead to erroneous conclusions. In that sense, an “objective index” of the changes occurring after CXL (or after any other surgery or external damage) is one of the keys to understand changes in the corneal morphology that cannot be seen by simple observation.

Quantitative tools such as the FFT [26, 29, 31] or a roughness score [27] have been shown to be useful procedures to detect changes in porcine corneal stroma after CXL treatment in both forward and backward SHG images. Similar to the results here obtained, the later configuration reported that standard CXL produced significant architectural changes of the deeper stroma.

In the conventional CXL treatment here used, the cornea is firstly deepithelialized. Then, during UVA radiation, stromal cells are killed (i.e., depletion areas appear). This was also reported in post-CXL porcine corneas by Gupta et al. [27]. During the following weeks, the healing process is responsible for the epithelial restoration [3] and the stromal regeneration. This stromal recovery needs, as any other wound healing operation [38, 54], several cellular divisions which produce activated keratocytes (bigger cells than regular keratocytes with more organelles) [25].

The histological analysis here presented corroborates that the CXL treatment covered the entire stroma of both animal models [2, 36]. However, unlike SHG imaging, this technique is unable to demonstrate the reorganization of the fibers. After the damage caused by the UVA radiation, the repopulation of cells takes place across the stroma, although this was slower in chickens than in rabbits. While CXL rabbit sections displayed larger and more heavily stained cells, chicken corneal sections displayed incomplete restoration with a band free of keratocytes (depletion region) in the anterior stroma.

In summary, the ST applied to SHG images of corneas represents a quantitative and accurate approach to objectively describe the degree of organization of the stromal architecture. Our findings show that the overall organization of corneas presenting a well-organized collagen assembly (such as the adult chicken or any other bird) is hardly affected by CXL treatment. On the contrary, there is a significant increase in collagen organization in CXL-treated corneas whose initial distribution is partially organized or nonorganized (such as the rabbit cornea). A paradigmatic example of a nonorganized collagen arrangement is a keratoconic cornea [18–22]. At this point, further experiments on the effects of CXL treatments in keratoconic human donor corneas will help to go a step further into the understanding of CXL effects at microscopic levels.

Although during the last 20 years SHG imaging of the cornea has been a powerful tool to explore the structure of the stroma under very different experimental conditions, those experiments were carried out in ex-vivo conditions. Measurements in living human eyes were always thought to be very challenging. However, we have recently reported in vivo SHG imaging of the human cornea for the very first time [55]. This demonstrates that SHG images of the living human eye can be safely acquired without photo-damage side effects. That compact clinical SHG instrument represents a promising ophthalmological tool and might easily be implemented into the clinics. It will undoubtedly improve the accuracy of diagnoses and will lead to a better early detection of corneal pathologies. The monitoring of diseased and surgically altered corneas could also benefit from this.

## Data Availability

The datasets used within this paper are available from the corresponding author upon reasonable request.
